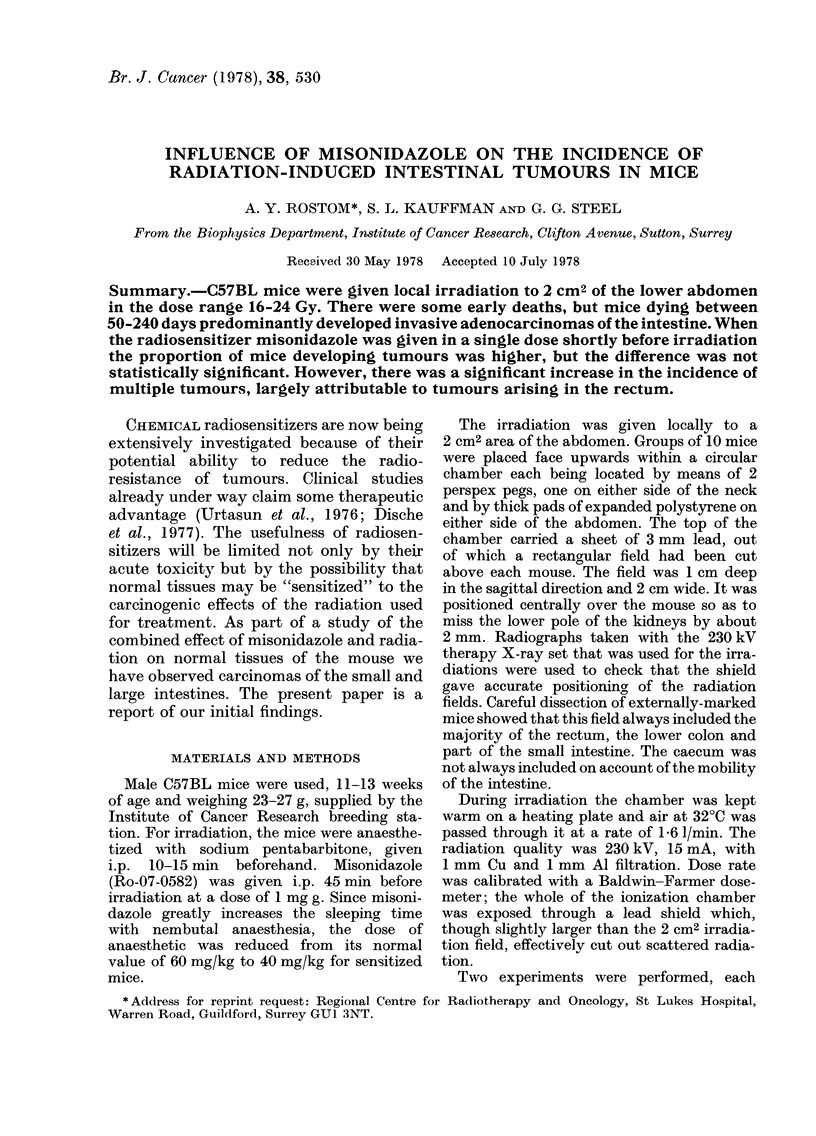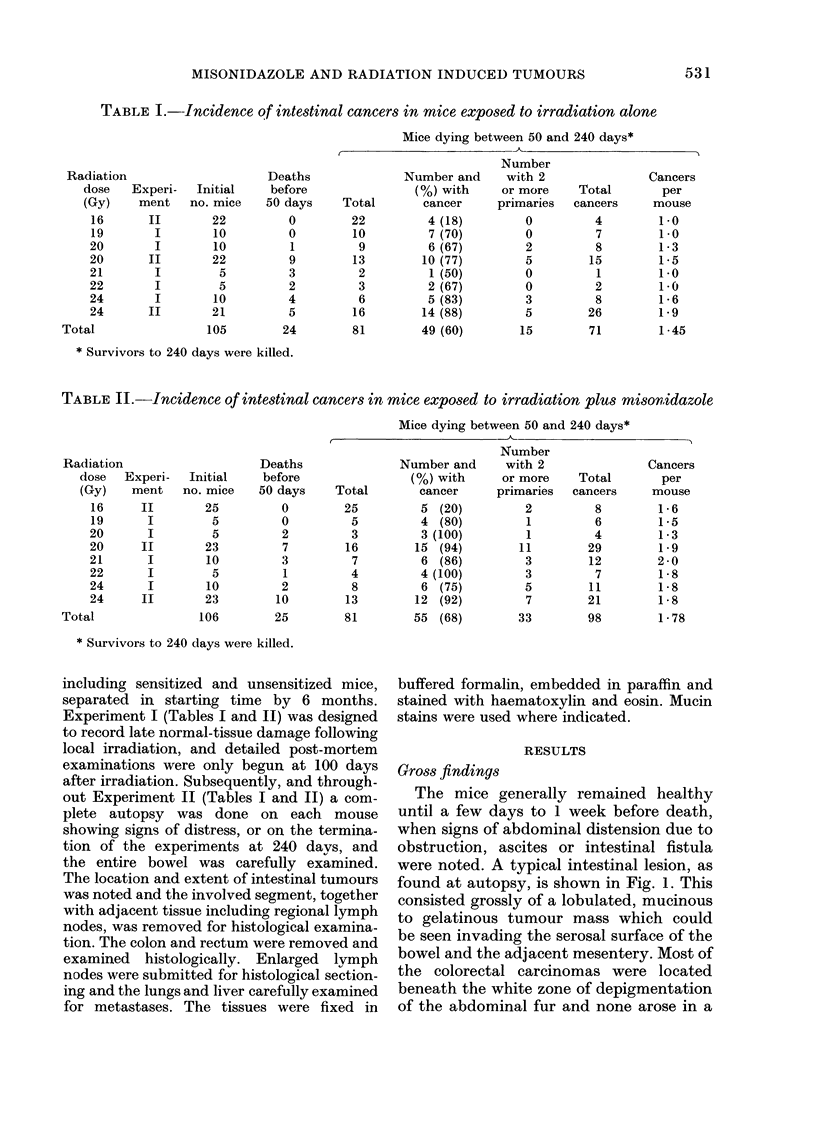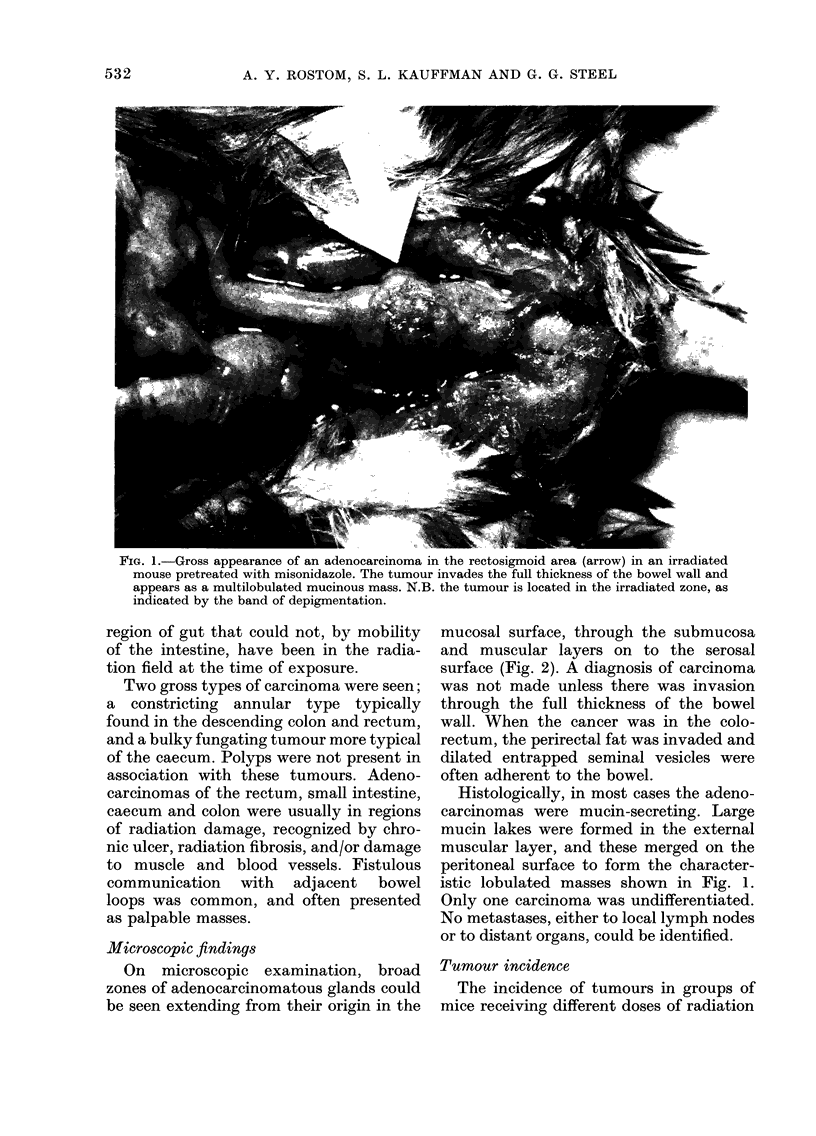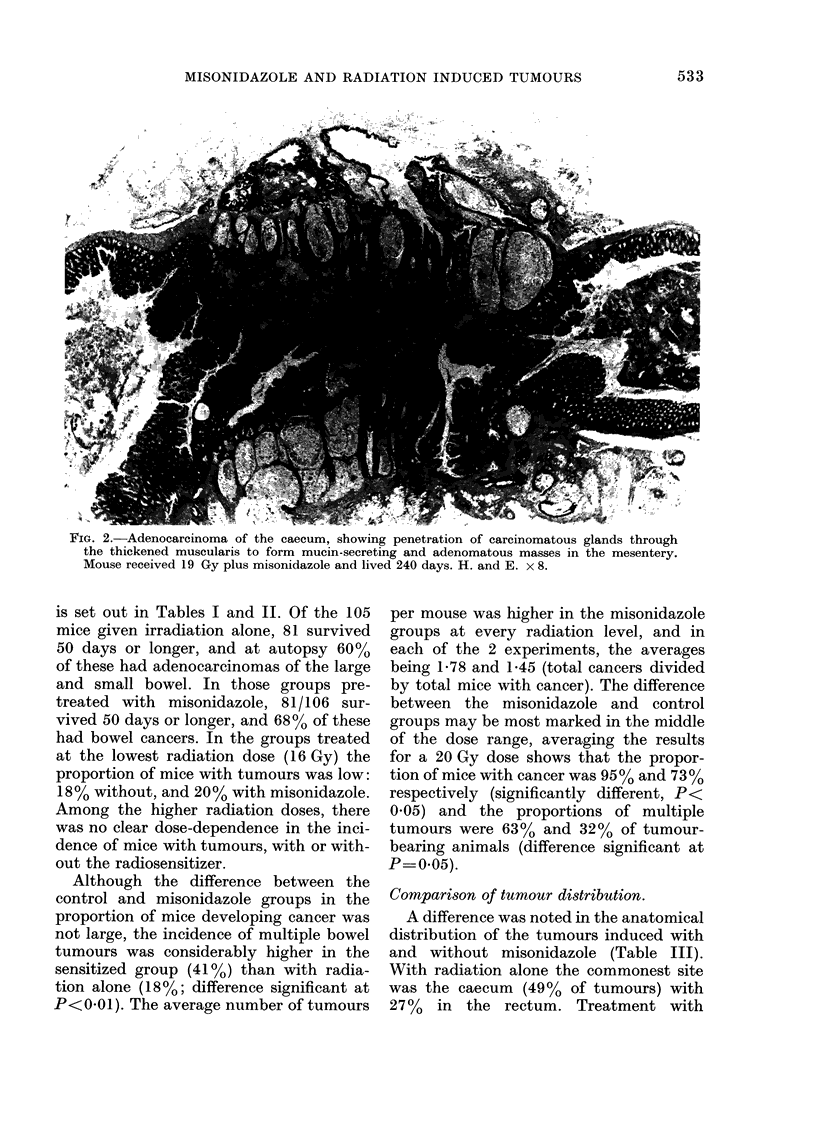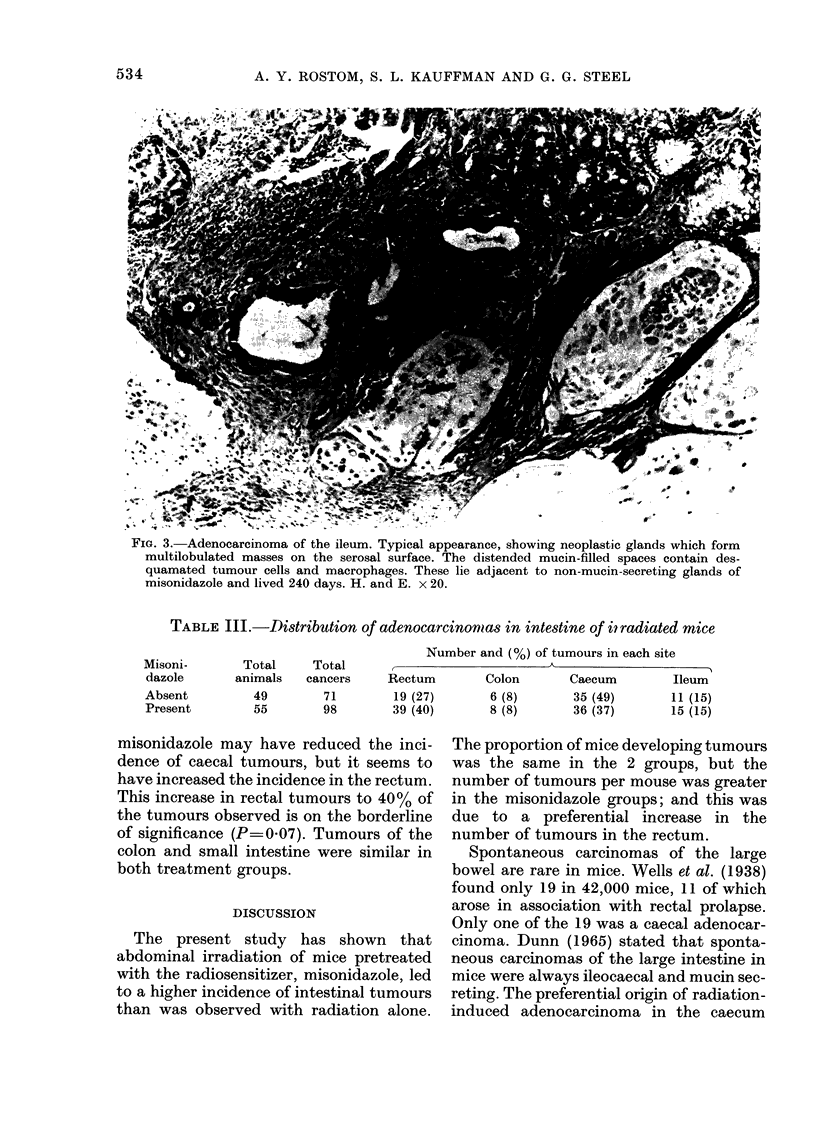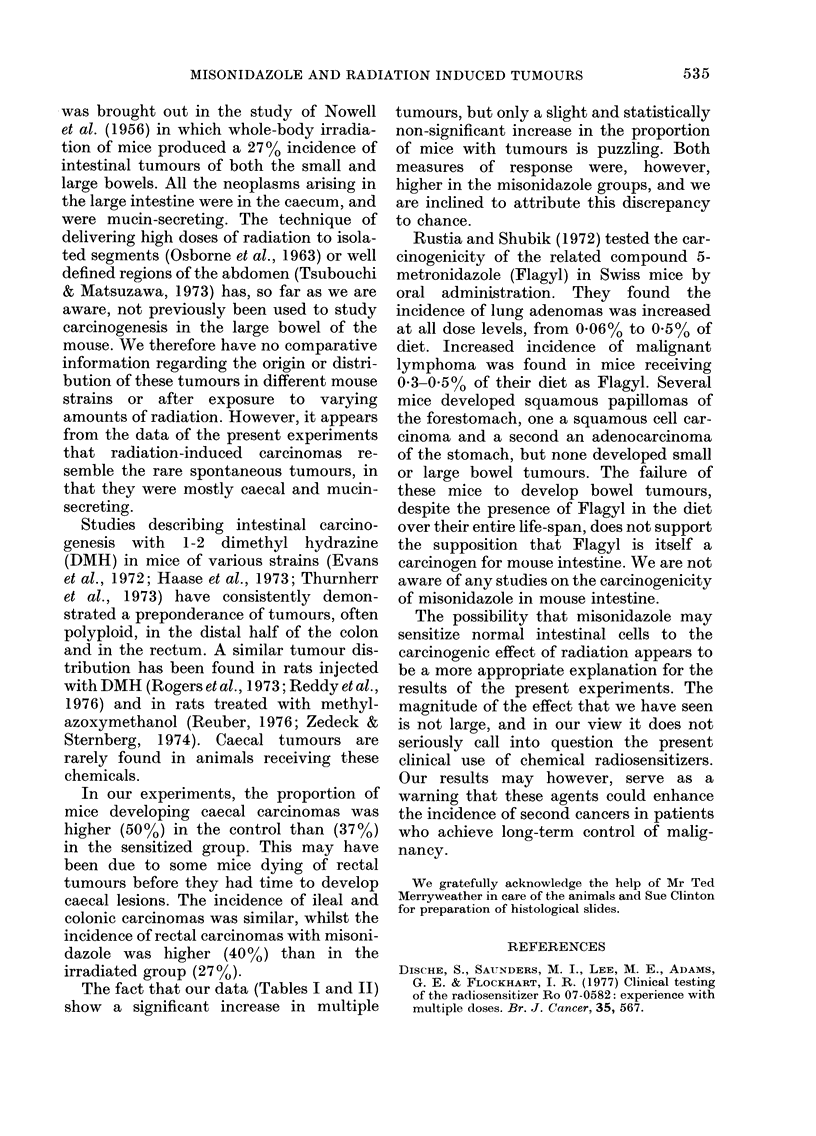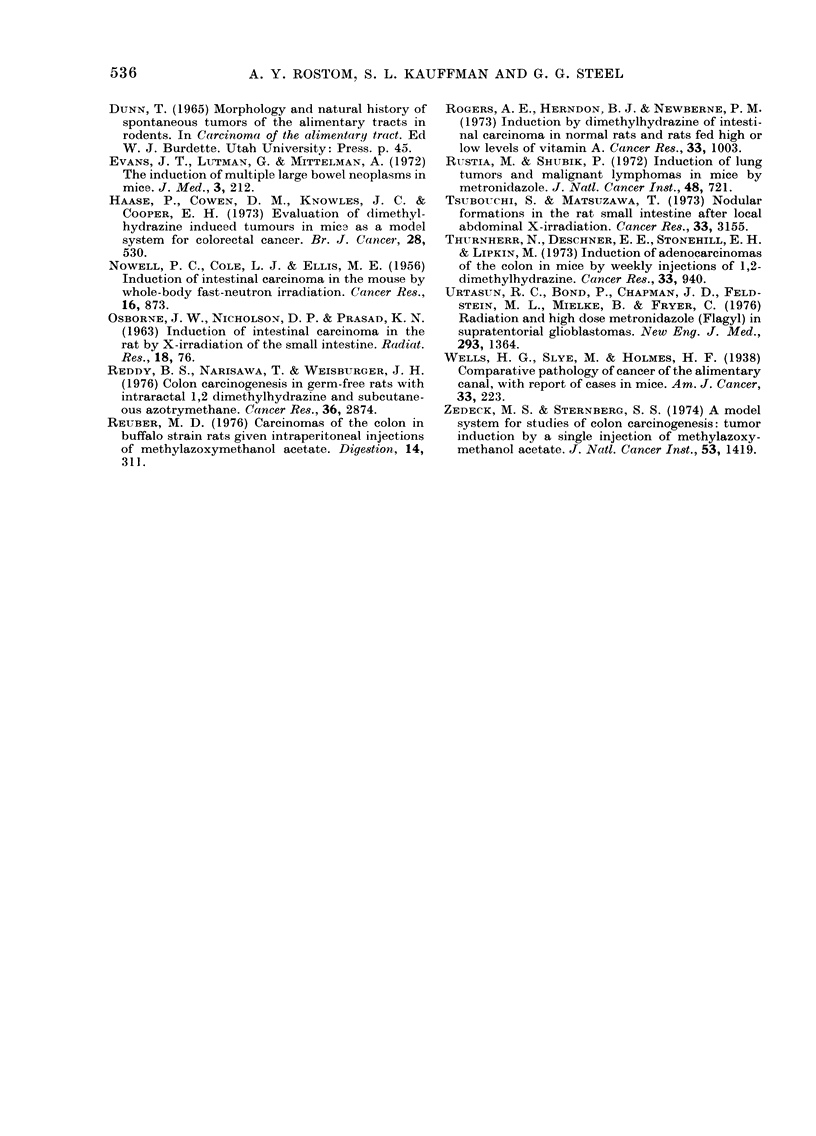# Influence of misonidazole on the incidence of radiation-induced intestinal tumours in mice.

**DOI:** 10.1038/bjc.1978.240

**Published:** 1978-10

**Authors:** A. Y. Rostom, S. L. Kauffman, G. G. Steel

## Abstract

**Images:**


*Br. J. Cancer* (1978), **38**, 530

### INFLUENCE OF MISONIDAZOLE ON THE INCIDENCE OF RADIATION-INDUCED INTESTINAL TUMOURS IN MICE

A. Y. ROSTOM*, S. L. KAUFFMAN AND G. G. STEEL

*From the Biophysics Department, Institute of Cancer Research, Clifton Avenue, Sutton, Surrey*

Received 30 May 1978 Accepted 10 July 1978

**Summary.—C57BL mice were given local irradiation to 2 cm² of the lower abdomen in the dose range 16-24 Gy. There were some early deaths, but mice dying between 50-240 days predominantly developed invasive adenocarcinomas of the intestine. When the radiosensitizer misonidazole was given in a single dose shortly before irradiation the proportion of mice developing tumours was higher, but the difference was not statistically significant. However, there was a significant increase in the incidence of multiple tumours, largely attributable to tumours arising in the rectum.**

CHEMICAL radiosensitizers are now being extensively investigated because of their potential ability to reduce the radioresistance of tumours. Clinical studies already under way claim some therapeutic advantage (Urtasun *et al.*, 1976; Dische *et al.*, 1977). The usefulness of radiosensitizers will be limited not only by their acute toxicity but by the possibility that normal tissues may be "sensitized" to the carcinogenic effects of the radiation used for treatment. As part of a study of the combined effect of misonidazole and radiation on normal tissues of the mouse we have observed carcinomas of the small and large intestines. The present paper is a report of our initial findings.

### MATERIALS AND METHODS

Male C57BL mice were used, 11–13 weeks of age and weighing 23–27 g, supplied by the Institute of Cancer Research breeding station. For irradiation, the mice were anaesthetized with sodium pentabarbitone, given i.p. 10–15 min beforehand. Misonidazole (Ro-07-0582) was given i.p. 45 min before irradiation at a dose of 1 mg g. Since misonidazole greatly increases the sleeping time with nembutal anaesthesia, the dose of anaesthetic was reduced from its normal value of 60 mg/kg to 40 mg/kg for sensitized mice.

The irradiation was given locally to a 2 cm² area of the abdomen. Groups of 10 mice were placed face upwards within a circular chamber each being located by means of 2 perspex pegs, one on either side of the neck and by thick pads of expanded polystyrene on either side of the abdomen. The top of the chamber carried a sheet of 3 mm lead, out of which a rectangular field had been cut above each mouse. The field was 1 cm deep in the sagittal direction and 2 cm wide. It was positioned centrally over the mouse so as to miss the lower pole of the kidneys by about 2 mm. Radiographs taken with the 230 kV therapy X-ray set that was used for the irradiations were used to check that the shield gave accurate positioning of the radiation fields. Careful dissection of externally-marked mice showed that this field always included the majority of the rectum, the lower colon and part of the small intestine. The caecum was not always included on account of the mobility of the intestine.

During irradiation the chamber was kept warm on a heating plate and air at 32°C was passed through it at a rate of 1·6 l/min. The radiation quality was 230 kV, 15 mA, with 1 mm Cu and 1 mm Al filtration. Dose rate was calibrated with a Baldwin–Farmer dosemeter; the whole of the ionization chamber was exposed through a lead shield which, though slightly larger than the 2 cm² irradiation field, effectively cut out scattered radiation.

Two experiments were performed, each

*Address for reprint request: Regional Centre for Radiotherapy and Oncology, St Lukes Hospital, Warren Road, Guildford, Surrey GU1 3NT.

TABLE I.—*Incidence of intestinal cancers in mice exposed to irradiation alone*

| | | | | Mice dying between 50 and 240 days* | | | | |
|---|---|---|---|---|---|---|---|---|
| Radiation dose (Gy) | Experiment | Initial no. mice | Deaths before 50 days | Total | Number and (%) with cancer | Number with 2 or more primaries | Total cancers | Cancers per mouse |
| 16 | II | 22 | 0 | 22 | 4 (18) | 0 | 4 | 1·0 |
| 19 | I | 10 | 0 | 10 | 7 (70) | 0 | 7 | 1·0 |
| 20 | I | 10 | 1 | 9 | 6 (67) | 2 | 8 | 1·3 |
| 20 | II | 22 | 9 | 13 | 10 (77) | 5 | 15 | 1·5 |
| 21 | I | 5 | 3 | 2 | 1 (50) | 0 | 1 | 1·0 |
| 22 | I | 5 | 2 | 3 | 2 (67) | 0 | 2 | 1·0 |
| 24 | I | 10 | 4 | 6 | 5 (83) | 3 | 8 | 1·6 |
| 24 | II | 21 | 5 | 16 | 14 (88) | 5 | 26 | 1·9 |
| Total | | 105 | 24 | 81 | 49 (60) | 15 | 71 | 1·45 |

* Survivors to 240 days were killed.

TABLE II.—*Incidence of intestinal cancers in mice exposed to irradiation plus misonidazole*

| | | | | Mice dying between 50 and 240 days* | | | | |
|---|---|---|---|---|---|---|---|---|
| Radiation dose (Gy) | Experiment | Initial no. mice | Deaths before 50 days | Total | Number and (%) with cancer | Number with 2 or more primaries | Total cancers | Cancers per mouse |
| 16 | II | 25 | 0 | 25 | 5 (20) | 2 | 8 | 1·6 |
| 19 | I | 5 | 0 | 5 | 4 (80) | 1 | 6 | 1·5 |
| 20 | I | 5 | 2 | 3 | 3 (100) | 1 | 4 | 1·3 |
| 20 | II | 23 | 7 | 16 | 15 (94) | 11 | 29 | 1·9 |
| 21 | I | 10 | 3 | 7 | 6 (86) | 3 | 12 | 2·0 |
| 22 | I | 5 | 1 | 4 | 4 (100) | 3 | 7 | 1·8 |
| 24 | I | 10 | 2 | 8 | 6 (75) | 5 | 11 | 1·8 |
| 24 | II | 23 | 10 | 13 | 12 (92) | 7 | 21 | 1·8 |
| Total | | 106 | 25 | 81 | 55 (68) | 33 | 98 | 1·78 |

* Survivors to 240 days were killed.

including sensitized and unsensitized mice, separated in starting time by 6 months. Experiment I (Tables I and II) was designed to record late normal-tissue damage following local irradiation, and detailed post-mortem examinations were only begun at 100 days after irradiation. Subsequently, and throughout Experiment II (Tables I and II) a complete autopsy was done on each mouse showing signs of distress, or on the termination of the experiments at 240 days, and the entire bowel was carefully examined. The location and extent of intestinal tumours was noted and the involved segment, together with adjacent tissue including regional lymph nodes, was removed for histological examination. The colon and rectum were removed and examined histologically. Enlarged lymph nodes were submitted for histological sectioning and the lungs and liver carefully examined for metastases. The tissues were fixed in buffered formalin, embedded in paraffin and stained with haematoxylin and eosin. Mucin stains were used where indicated.

### RESULTS

### *Gross findings*

The mice generally remained healthy until a few days to 1 week before death, when signs of abdominal distension due to obstruction, ascites or intestinal fistula were noted. A typical intestinal lesion, as found at autopsy, is shown in Fig. 1. This consisted grossly of a lobulated, mucinous to gelatinous tumour mass which could be seen invading the serosal surface of the bowel and the adjacent mesentery. Most of the colorectal carcinomas were located beneath the white zone of depigmentation of the abdominal fur and none arose in a

FIG. 1.—Gross appearance of an adenocarcinoma in the rectosigmoid area (arrow) in an irradiated mouse pretreated with misonidazole. The tumour invades the full thickness of the bowel wall and appears as a multilobulated mucinous mass. N.B. the tumour is located in the irradiated zone, as indicated by the band of depigmentation.

region of gut that could not, by mobility of the intestine, have been in the radiation field at the time of exposure.

Two gross types of carcinoma were seen; a constricting annular type typically found in the descending colon and rectum, and a bulky fungating tumour more typical of the caecum. Polyps were not present in association with these tumours. Adenocarcinomas of the rectum, small intestine, caecum and colon were usually in regions of radiation damage, recognized by chronic ulcer, radiation fibrosis, and/or damage to muscle and blood vessels. Fistulous communication with adjacent bowel loops was common, and often presented as palpable masses.

*Microscopic findings*

On microscopic examination, broad zones of adenocarcinomatous glands could be seen extending from their origin in the mucosal surface, through the submucosa and muscular layers on to the serosal surface (Fig. 2). A diagnosis of carcinoma was not made unless there was invasion through the full thickness of the bowel wall. When the cancer was in the colorectum, the perirectal fat was invaded and dilated entrapped seminal vesicles were often adherent to the bowel.

Histologically, in most cases the adenocarcinomas were mucin-secreting. Large mucin lakes were formed in the external muscular layer, and these merged on the peritoneal surface to form the characteristic lobulated masses shown in Fig. 1. Only one carcinoma was undifferentiated. No metastases, either to local lymph nodes or to distant organs, could be identified.

*Tumour incidence*

The incidence of tumours in groups of mice receiving different doses of radiation

FIG. 2.—Adenocarcinoma of the caecum, showing penetration of carcinomatous glands through the thickened muscularis to form mucin-secreting and adenomatous masses in the mesentery. Mouse received 19 Gy plus misonidazole and lived 240 days. H. and E. ×8.

is set out in Tables I and II. Of the 105 mice given irradiation alone, 81 survived 50 days or longer, and at autopsy 60% of these had adenocarcinomas of the large and small bowel. In those groups pretreated with misonidazole, 81/106 survived 50 days or longer, and 68% of these had bowel cancers. In the groups treated at the lowest radiation dose (16 Gy) the proportion of mice with tumours was low: 18% without, and 20% with misonidazole. Among the higher radiation doses, there was no clear dose-dependence in the incidence of mice with tumours, with or without the radiosensitizer.

Although the difference between the control and misonidazole groups in the proportion of mice developing cancer was not large, the incidence of multiple bowel tumours was considerably higher in the sensitized group (41%) than with radiation alone (18%; difference significant at $P<0{\cdot}01$). The average number of tumours per mouse was higher in the misonidazole groups at every radiation level, and in each of the 2 experiments, the averages being 1·78 and 1·45 (total cancers divided by total mice with cancer). The difference between the misonidazole and control groups may be most marked in the middle of the dose range, averaging the results for a 20 Gy dose shows that the proportion of mice with cancer was 95% and 73% respectively (significantly different, $P<0{\cdot}05$) and the proportions of multiple tumours were 63% and 32% of tumour-bearing animals (difference significant at $P=0{\cdot}05$).

*Comparison of tumour distribution.*

A difference was noted in the anatomical distribution of the tumours induced with and without misonidazole (Table III). With radiation alone the commonest site was the caecum (49% of tumours) with 27% in the rectum. Treatment with

FIG. 3.—Adenocarcinoma of the ileum. Typical appearance, showing neoplastic glands which form multilobulated masses on the serosal surface. The distended mucin-filled spaces contain desquamated tumour cells and macrophages. These lie adjacent to non-mucin-secreting glands of misonidazole and lived 240 days. H. and E. × 20.

TABLE III.—*Distribution of adenocarcinomas in intestine of irradiated mice*

| Misonidazole | Total animals | Total cancers | Number and (%) of tumours in each site | | | |
|---|---|---|---|---|---|---|
| | | | Rectum | Colon | Caecum | Ileum |
| Absent | 49 | 71 | 19 (27) | 6 (8) | 35 (49) | 11 (15) |
| Present | 55 | 98 | 39 (40) | 8 (8) | 36 (37) | 15 (15) |

misonidazole may have reduced the incidence of caecal tumours, but it seems to have increased the incidence in the rectum. This increase in rectal tumours to 40% of the tumours observed is on the borderline of significance ($P=0{\cdot}07$). Tumours of the colon and small intestine were similar in both treatment groups.

### DISCUSSION

The present study has shown that abdominal irradiation of mice pretreated with the radiosensitizer, misonidazole, led to a higher incidence of intestinal tumours than was observed with radiation alone. The proportion of mice developing tumours was the same in the 2 groups, but the number of tumours per mouse was greater in the misonidazole groups; and this was due to a preferential increase in the number of tumours in the rectum.

Spontaneous carcinomas of the large bowel are rare in mice. Wells *et al.* (1938) found only 19 in 42,000 mice, 11 of which arose in association with rectal prolapse. Only one of the 19 was a caecal adenocarcinoma. Dunn (1965) stated that spontaneous carcinomas of the large intestine in mice were always ileocaecal and mucin secreting. The preferential origin of radiation-induced adenocarcinoma in the caecum was brought out in the study of Nowell *et al.* (1956) in which whole-body irradiation of mice produced a 27% incidence of intestinal tumours of both the small and large bowels. All the neoplasms arising in the large intestine were in the caecum, and were mucin-secreting. The technique of delivering high doses of radiation to isolated segments (Osborne *et al.*, 1963) or well defined regions of the abdomen (Tsubouchi & Matsuzawa, 1973) has, so far as we are aware, not previously been used to study carcinogenesis in the large bowel of the mouse. We therefore have no comparative information regarding the origin or distribution of these tumours in different mouse strains or after exposure to varying amounts of radiation. However, it appears from the data of the present experiments that radiation-induced carcinomas resemble the rare spontaneous tumours, in that they were mostly caecal and mucin-secreting.

Studies describing intestinal carcinogenesis with 1-2 dimethyl hydrazine (DMH) in mice of various strains (Evans *et al.*, 1972; Haase *et al.*, 1973; Thurnherr *et al.*, 1973) have consistently demonstrated a preponderance of tumours, often polyploid, in the distal half of the colon and in the rectum. A similar tumour distribution has been found in rats injected with DMH (Rogers *et al.*, 1973; Reddy *et al.*, 1976) and in rats treated with methylazoxymethanol (Reuber, 1976; Zedeck & Sternberg, 1974). Caecal tumours are rarely found in animals receiving these chemicals.

In our experiments, the proportion of mice developing caecal carcinomas was higher (50%) in the control than (37%) in the sensitized group. This may have been due to some mice dying of rectal tumours before they had time to develop caecal lesions. The incidence of ileal and colonic carcinomas was similar, whilst the incidence of rectal carcinomas with misonidazole was higher (40%) than in the irradiated group (27%).

The fact that our data (Tables I and II) show a significant increase in multiple tumours, but only a slight and statistically non-significant increase in the proportion of mice with tumours is puzzling. Both measures of response were, however, higher in the misonidazole groups, and we are inclined to attribute this discrepancy to chance.

Rustia and Shubik (1972) tested the carcinogenicity of the related compound 5-metronidazole (Flagyl) in Swiss mice by oral administration. They found the incidence of lung adenomas was increased at all dose levels, from 0·06% to 0·5% of diet. Increased incidence of malignant lymphoma was found in mice receiving 0·3–0·5% of their diet as Flagyl. Several mice developed squamous papillomas of the forestomach, one a squamous cell carcinoma and a second an adenocarcinoma of the stomach, but none developed small or large bowel tumours. The failure of these mice to develop bowel tumours, despite the presence of Flagyl in the diet over their entire life-span, does not support the supposition that Flagyl is itself a carcinogen for mouse intestine. We are not aware of any studies on the carcinogenicity of misonidazole in mouse intestine.

The possibility that misonidazole may sensitize normal intestinal cells to the carcinogenic effect of radiation appears to be a more appropriate explanation for the results of the present experiments. The magnitude of the effect that we have seen is not large, and in our view it does not seriously call into question the present clinical use of chemical radiosensitizers. Our results may however, serve as a warning that these agents could enhance the incidence of second cancers in patients who achieve long-term control of malignancy.

We gratefully acknowledge the help of Mr Ted Merryweather in care of the animals and Sue Clinton for preparation of histological slides.

### REFERENCES

Dische, S., Saunders, M. I., Lee, M. E., Adams, G. E. & Flockhart, I. R. (1977) Clinical testing of the radiosensitizer Ro 07-0582: experience with multiple doses. *Br. J. Cancer*, **35**, 567.

DUNN, T. (1965) Morphology and natural history of spontaneous tumors of the alimentary tracts in rodents. In *Carcinoma of the alimentary tract*. Ed W. J. Burdette. Utah University: Press. p. 45.

EVANS, J. T., LUTMAN, G. & MITTELMAN, A. (1972) The induction of multiple large bowel neoplasms in mice. *J. Med.*, **3**, 212.

HAASE, P., COWEN, D. M., KNOWLES, J. C. & COOPER, E. H. (1973) Evaluation of dimethylhydrazine induced tumours in mice as a model system for colorectal cancer. *Br. J. Cancer*, **28**, 530.

NOWELL, P. C., COLE, L. J. & ELLIS, M. E. (1956) Induction of intestinal carcinoma in the mouse by whole-body fast-neutron irradiation. *Cancer Res.*, **16**, 873.

OSBORNE, J. W., NICHOLSON, D. P. & PRASAD, K. N. (1963) Induction of intestinal carcinoma in the rat by X-irradiation of the small intestine. *Radiat. Res.*, **18**, 76.

REDDY, B. S., NARISAWA, T. & WEISBURGER, J. H. (1976) Colon carcinogenesis in germ-free rats with intraractal 1,2 dimethylhydrazine and subcutaneous azotrymethane. *Cancer Res.*, **36**, 2874.

REUBER, M. D. (1976) Carcinomas of the colon in buffalo strain rats given intraperitoneal injections of methylazoxymethanol acetate. *Digestion*, **14**, 311.

ROGERS, A. E., HERNDON, B. J. & NEWBERNE, P. M. (1973) Induction by dimethylhydrazine of intestinal carcinoma in normal rats and rats fed high or low levels of vitamin A. *Cancer Res.*, **33**, 1003.

RUSTIA, M. & SHUBIK, P. (1972) Induction of lung tumors and malignant lymphomas in mice by metronidazole. *J. Natl. Cancer Inst.*, **48**, 721.

TSUBOUCHI, S. & MATSUZAWA, T. (1973) Nodular formations in the rat small intestine after local abdominal X-irradiation. *Cancer Res.*, **33**, 3155.

THURNHERR, N., DESCHNER, E. E., STONEHILL, E. H. & LIPKIN, M. (1973) Induction of adenocarcinomas of the colon in mice by weekly injections of 1,2-dimethylhydrazine. *Cancer Res.*, **33**, 940.

URTASUN, R. C., BOND, P., CHAPMAN, J. D., FELDSTEIN, M. L., MIELKE, B. & FRYER, C. (1976) Radiation and high dose metronidazole (Flagyl) in supratentorial glioblastomas. *New Eng. J. Med.*, **293**, 1364.

WELLS, H. G., SLYE, M. & HOLMES, H. F. (1938) Comparative pathology of cancer of the alimentary canal, with report of cases in mice. *Am. J. Cancer*, **33**, 223.

ZEDECK, M. S. & STERNBERG, S. S. (1974) A model system for studies of colon carcinogenesis: tumor induction by a single injection of methylazoxymethanol acetate. *J. Natl. Cancer Inst.*, **53**, 1419.